# A Participatory Design Approach to Develop Visualization of Wearable Actigraphy Data for Health Care Professionals: Case Study in Qatar

**DOI:** 10.2196/25880

**Published:** 2022-04-08

**Authors:** Kamran Khowaja, Wafa Waheeda Syed, Meghna Singh, Shahrad Taheri, Odette Chagoury, Dena Al-Thani, Michaël Aupetit

**Affiliations:** 1 Information and Computing Technology Division College of Science and Engineering Hamad Bin Khalifa University Education City Qatar; 2 Department of Computer Science Shaheed Zulfikar Ali Bhutto Institute of Science and Technology Hyderabad Pakistan; 3 Social Computing, Qatar Computing Research Institute Hamad Bin Khalifa University Education City Qatar; 4 Department of Computer Science and Engineering University of Minnesota Minneapolis, MN United States; 5 Department of Medicine Weill Cornell Medicine Doha Qatar; 6 Department of Medicine Weill Cornell Medicine New York, NY United States; 7 National Obesity Treatment Center Qatar Metabolic Institute Doha Qatar

**Keywords:** participatory design, user-centered design, visualization, health care professional, persona, brainwriting, heuristic walkthrough, use case, interface walkthrough

## Abstract

**Background:**

Several tools have been developed for health care professionals to monitor the physical activity of their patients, but most of these tools have been considering only the needs of users in North American and European countries and applicable for only specific analytic tasks. To our knowledge, no research study has utilized the participatory design (PD) approach in the Middle East region to develop such tools, involving all the stakeholders in the product development phases, and no clear use cases have been derived from such studies that could serve future development in the field.

**Objective:**

This study aims to develop an interactive visualization tool (ActiVis) to support local health care professionals in monitoring the physical activity of their patients measured through wearable sensors, with the overall objective of improving the health of the Qatari population.

**Methods:**

We used PD and user-centered design methodologies to develop ActiVis, including persona development, brainwriting, and heuristic walkthrough as part of user evaluation workshops; and use cases, heuristic walkthrough, interface walkthrough, and survey as part of expert evaluation sessions.

**Results:**

We derived and validated 6 data analysis use cases targeted at specific health care professionals from a collaborative design workshop and an expert user study. These use cases led to improving the design of the ActiVis tool to support the monitoring of patients’ physical activity by nurses and family doctors. The ActiVis research prototype (RP) compared favorably with the Fitbit Dashboard, showing the importance of design tools specific to end users’ needs rather than relying on repurposing existing tools designed for other types of users. The use cases we derived happen to be culturally agnostic, despite our assumption that the local Muslim and Arabic culture could impact the design of such visualization tools. At last, taking a step back, we reflect on running collaborative design sessions in a multicultural environment and oil-based economy.

**Conclusions:**

Beyond the development of the ActiVis tool, this study can serve other visualization and human–computer interaction designers in the region to prepare their design projects and encourage health care professionals to engage with designers and engineers to improve the tools they use for supporting their daily routine. The development of the ActiVis tool for nurses, and other visualization tools specific to family doctors and clinician researchers, is still ongoing and we plan to integrate them into an operational platform for health care professionals in Qatar in the near future.

## Introduction

According to the World Health Organization (WHO) report of 2018 [1,2], lack of physical activity is the fourth leading risk factor for mortality. Physical activity reduces the risk of coronary heart disease, stroke, hypertension, depression, type 2 diabetes, and several types of cancer. Unfortunately, physical activity across many countries is declining. In the context of Qatar, researchers at Weill Cornell Medicine - Qatar (WCM-Q) conducted a study among elementary school children between ages 7 and 12 [3]. The authors found that 42.1% of these children were either obese or overweight, and their sleep was significantly shorter than children with a healthy weight. In another study on prevalent health issues among Qatari citizens and long-term residents [4], the authors found that 83% of the population undertook little to no physical activity, and almost half of the population did not do any physical exercise. Hence, there is a need to increase the physical activity of the Qatar population to reduce the risk of related diseases as mentioned in the WHO 2018 report.

Many behavioral modification programs have been developed for more than 2 decades to reduce physical inactivity [5-8]. Nowadays, technologies allow continuous recording of individual physical activity over several days. Moreover, the use of smartphones and wearable devices (smartwatches, wristbands, etc) among children, adults, or older adults has increased in the last decade. Smartphones and wearable devices are then actively used to record, measure, and monitor body movement and activities performed by an individual using global positioning system and accelerometer installed on these devices [1,9-11]. The visualization of the recorded activity data can then show the time when an individual was the most or least active throughout the day [12] and support monitoring and exploration of such activities. We focus on the design of such visualization tools in this work.

There is a growing trend in visualization studies to explore ways to represent wearable data for self-monitoring sleep [13], analyze data by health coaches [14] or researchers [15], evaluate performance dashboard for sport [16], or evaluate time-based activity graphical representations on mobile phones [17]. Some other studies explored the best approach to visualizing the data to support behavior change [18,19] or provided a visualization dashboard to help patients understand their longitudinal health data [20]. Still, the visualization of wearable data is an active research area. A natural approach to start with is to repurpose existing visualization tools such as the Fitbit Dashboard to visualize data in a health care setting, but the actual needs of health care professionals may depart substantially from the ones of the general public self-tracking their physical activities. To the best of our knowledge, there is no visualization tool specifically designed to support the health care professionals in monitoring and analyzing physical activities of patients through their wearable actigraph data. We also could not find a set of use cases and user roles covering such needs.

Moreover, several studies [21-23] have demonstrated the importance of the cultural, social, and local context when designing medical or health care technological solutions. Despite this view, the literature on technology acceptance mostly concentrates on highly developed North American and European countries, and little is known about health technology use and data visualization in the Arab world, including the Gulf countries [24-29] such as Qatar [3,4]. Arab countries share lots of similarities, such as cultural and religious values, language, and lifestyle [30,31], and are quite different from North American and European countries. Salgado et al [32] has highlighted that culture plays a vital role from the investigation to the design or development of new methods, theories, techniques, and systems. Hence, cultural specificities were expected when we started this project and we decided to follow a participatory design (PD) approach to collect the potentially culturally specific needs of end users.

Alabdulqader et al [33] highlighted a need to reduce the cultural gap between technology designers and users by using a PD approach. PD aims to design solutions that consider the local context and culture and has been used effectively in the health/medical domain [34-44]. PD allows researchers to involve potential users of a product or technological solution in the ideation, design, development, or appropriation of the solution [35]. Kanstrup et al [35], as a part of their review, found that workshop/group sessions/focus groups, interviews, and prototyping have been more commonly used in PD sessions of health information technology. We followed this approach in our studies.

The use of opportunistic research and sampling is commonly used in health care research as it allows researchers to use the available participants or research instruments to perform research chores [45-49]. To the best of our knowledge, there is no interactive tool that has considered the needs of local health care professionals in Qatar in their regular activities. These activities include understanding and monitoring their patients and helping/assisting them to improve their physical activity, sleep, and eventually reduce obesity. Results from a previous study [4], informal discussion with the authors [3], and an approach of opportunistic research were used as a basis to design the first prototype of an interactive tool (ActiVis) to support the mentioned needs of the local health care professionals.

This paper reports on the PD and summative evaluation of a second version of the ActiVis prototype to visualize activity data from wearable devices, which meets the needs of local health care professionals for monitoring the physical activity of their patients, to improve the physical activity of the Qatar population. We use methodologies from user-centered design [50-58] and PD for the first time to design eHealth data visualization in Qatar.

## Methods

### Research Protocol and User Studies

#### Overview

The users and their needs increased over time as studies were conducted as a part of this research and ActiVis was accordingly modified and reported in different sections of this research. The development of any technological solution is not an easy task. It requires gathering and analysis of considerable data from the ideation to the design, development, evaluation, and deployment of the technology. It becomes even more challenging when the local context needs to be considered and incorporated into the technology. The data collection and analysis methods vary from one study to another due to various constraints such as the availability of the target users and the initial uncertainty in the direction of the project, which is refined progressively through the development cycles.

**Figure 1:**
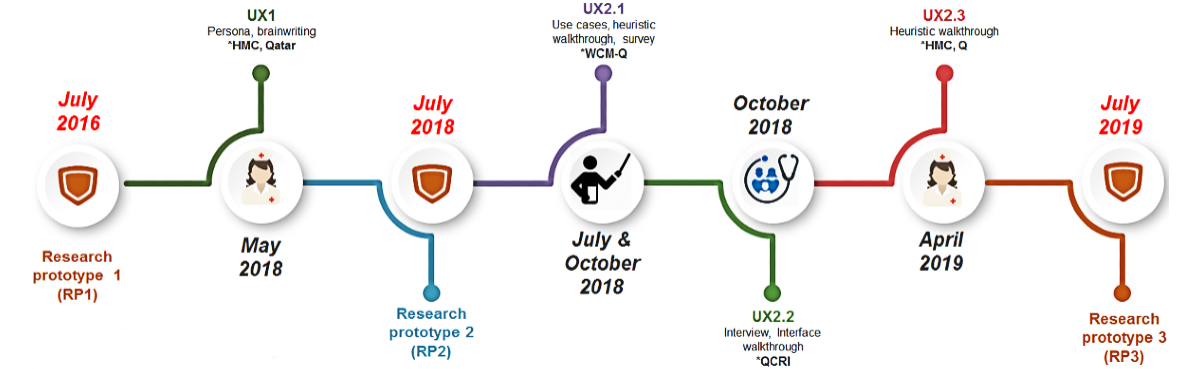
Research Prototypes (RP) designed and developed, and User Studies (UX) conducted throughout the project. HMC-Q: Hamad Medical Corporation - Qatar; WCM-Q: Weill Cornell Medicine - Qatar; QCRI: Qatar Computing Research Institute.

Figure 1 shows the timeline of this work, the studies conducted with their target audience, the methods used, and the venues where they took place. The RPs developed and the user studies (UX) conducted are reported in Textbox 1.

**Research protocols and user studies conducted.**

- RP1: The first research protocol (RP) of ActiVis was developed out of a previous design expert analysis of the requirements not reported here.
- UX1: The first user study (UX) was a workshop conducted with nurses at Hamad Medical Corporation (the largest public health care provider in Qatar) to gather detailed requirements, personas, and usage scenarios, to design and develop the second RP of ActiVis (RP2) together with a set of 6 use cases targeting health care professionals.
- RP2: A total of 3 UX (UX2.1-UX2.3) were conducted to evaluate RP2 on 3 of these use cases. Each study was targeted at 1 type of user as follows:
- UX2.1: First, an expert evaluation was conducted with clinical researchers at Weill Cornell Medicine - Qatar (WCM-Q). UX2.1 supported improving the descriptions of the use cases, determining which type of health care professional users among nurses, family doctors, and clinician researchers were the actual targets, and evaluating RP2 based on the use cases targeted at nurses and family doctors. Usability issues were also identified as a part of that study.
- UX2.2: Then, an expert evaluation was conducted with a family doctor visiting Qatar Computing Research Institute (QCRI) to evaluate the second prototype RP2 based on use cases specific to that role as identified from UX2.1.
- UX2.3: Lastly, a workshop was conducted with nurses from Hamad Medical Corporation. The purpose was to evaluate RP2 on the use case specifically targeted at nurses and to compare RP2 with the Fitbit Dashboard as it provided similar functionalities. The study would allow researchers to understand the differences between both dashboards from the participants’ perspective and improve ActiVis based on their feedback. In this study, Fitbit was used as a comparison because it has a well-thought design [59,60] with similar functionalities required to support the user tasks, and it was the leading wearable technology in the consumer market at the time of the study [61].
- RP3: These studies (UX2.1-UX2.3) led to the design specifications for a third RP not reported here.

The protocol of the studies is described in the remaining subsections, while the results of each study are presented in the “Results” section.

#### RP1: Visual Analytic Tool for Actigraphy Sensor Data

**Figure 2:**
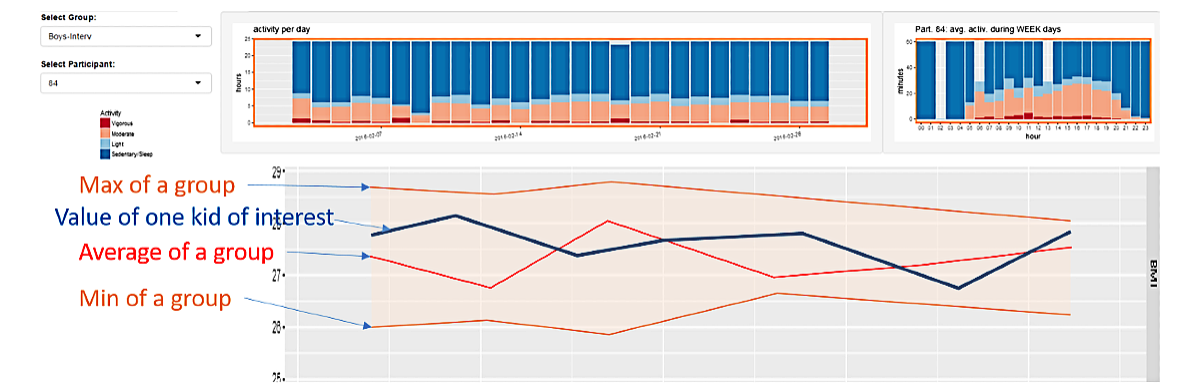
ActiVis research prototype 1.

In 2016, one of the authors (MA) started working on a visualization dashboard of wearable data for clinical decision making by health care professionals. This dashboard is aimed at supporting patients to move toward a healthier lifestyle based on their physical activity data. Figure 2 shows parts from the different screens of the initial visualization dashboard (ActiVis) developed as an RP (RP1) based on extensive discussions with health care professionals having expertise in childhood obesity and diabetes in Qatar. The data and initial user needs to be used to design the first prototype were collected as a part of a previous research project [62,63]. The details of RP1 reported in this paper are presented in the “RP1” subsection of the “Results” section.

#### UX1: Users’ Evaluation Workshop 1 With Nurses

##### Overview

A first user experiment (UX1) was conducted with the nursing staff of Hamad Medical Corporation on May 2, 2018. The workshop was conducted to gather some of the potential users to generate ideas for the prototype taking the local needs into account. The objective of the workshop was to learn about nurses’ perception of how visual analytics may enable them to promote lifestyle change and provide better advice to patients based on the activity data that would be collected from the patient’s wearable (smartwatch). The session was focused on patients with type 2 diabetes. It included a presentation followed by a brainwriting [64] session, where nurses in groups provided their input on desired information and computer technology solutions to support patient lifestyle changes.

##### Findings

Our analysis of the data collected from this study led to the design of 6 use cases, and the corresponding user tasks led to the technical specifications of the visualization design that we implemented in the second prototype (RP2) of ActiVis. It is to be noted that use cases were developed from the perspective of nurses who are one of the potential users of the ActiVis tool. However, it was not clear if the description of each use case was adequate or required some improvement, and if all the use cases would need to be implemented in the ActiVis tool, justifying the needs for another set of UX (UX2.1).

##### Participants

A total of 45 male and female participants, which included nurses as well as nursing informatics professionals working at Hamad Medical Corporation-Qatar (HMC-Q), attended the workshop.

##### Study Protocol

**Figure 3:**
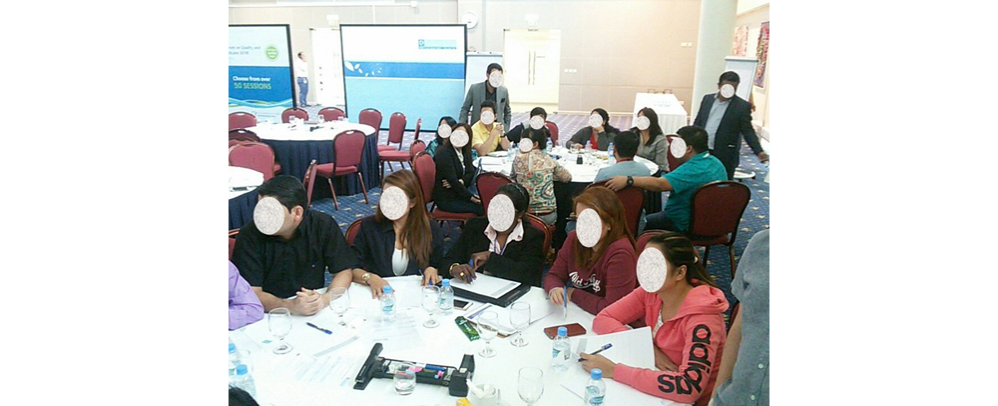
User study 1 workshop with nurses.

The nursing staff working at HMC-Q were recruited through an announcement by the chairperson of the nursing department, inviting them into the workshop as shown in Figure 3 to contribute to the development of the health care solution. The participants were split into 4 groups (10-12 members in each) for the brainwriting activities. Each group was provided with a flipchart and markers in addition to in-house designed templates and gamification cards to stimulate creativity and support groups in the brainwriting process. The brainwriting process involved 4 stages:

- Stage 1: Define a “Persona”—either a nurse or a patient with diabetes. The definition must include a short biography, goals, and objectives of the persona, as well as challenges and frustrations.
- Stage 2: Describe a typical scenario, either a single encounter for the nurse or a day in the patient’s life, highlighting issues and problems.
- Stage 3: Imagine the technologies that can help resolve the problems in the scenario considering the defined characteristics of the persona. Group members then vote for the best resolution.
- Stage 4: Rewrite the scenario in stage 2 including the best technology voted for in stage 3.

#### RP2: Research Prototype 2

**Figure 4:**
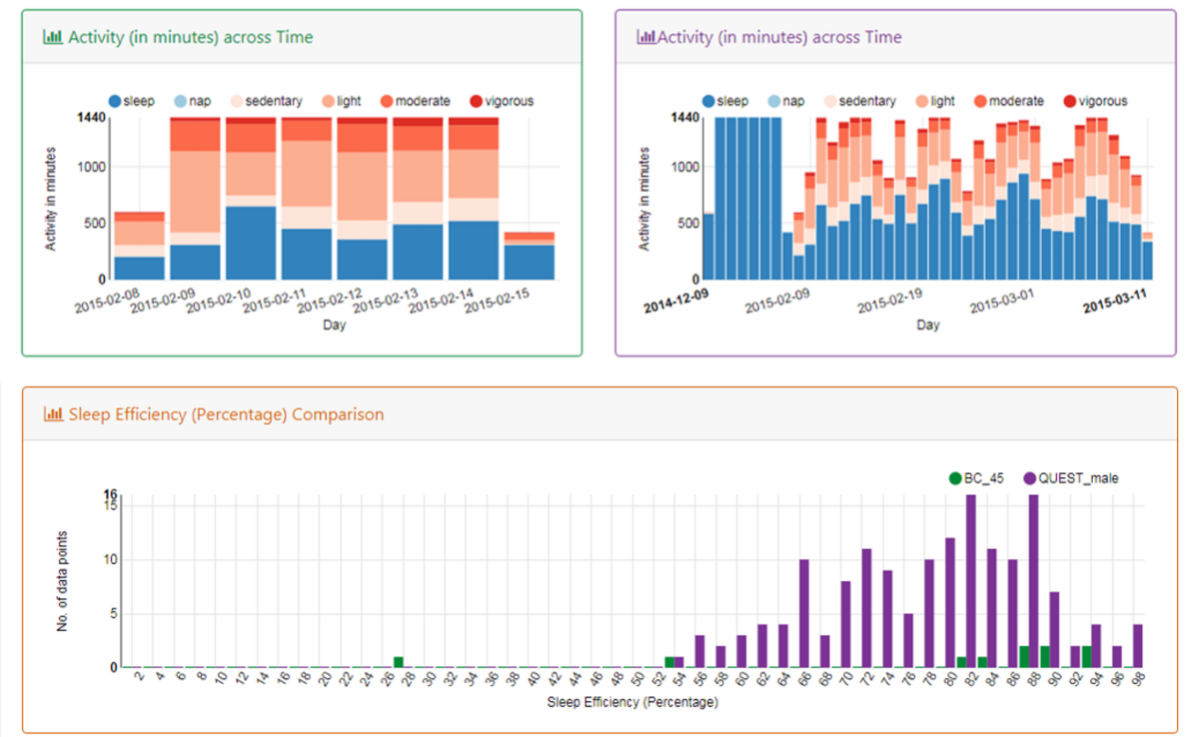
ActiVis research prototype 2.

The design of the second version of the ActiVis RP (RP2) was built on the use cases developed from the first user experiment UX1. Figure 4 shows parts from the different screens of the RP2 separated by a horizontal line while the details of RP2 are presented in the “Results” section.

#### UX2.1: Expert Evaluation 1

##### Overview

An expert review [65] study (UX2.1) of the second prototype (RP2) was conducted at WCQ-M. The expert review study included use cases, surveys, questionnaires, and heuristic walkthroughs. The WCM-Q group were invited for 2 studies.

##### UX2.1.1: Study 1

The participants went through the use cases [66,67] developed by the designers after analysis of the personas and usage scenarios from the first workshop (UX1) conducted with the nursing staff at HMC. Each participant was also asked to follow a think-aloud protocol when performing the task described in the use cases with the RP2 interface. Additional suggestions were provided toward the end of the evaluation in the survey questionnaire. The target users of the use cases were refined based on the suggestions from the participants.

##### UX2.1.2: Study 2

We used the heuristic walkthrough technique [68] to get participants ’ suggestions and improve the prototype further. The participants completed pre- and poststudy questionnaires as well. The identified usability problems were fixed before the updated version of the prototype was further evaluated in the following user experiments (UX2.2).

##### Participants

The participants were working in the area of diabetes research at WCM-Q. The demographic information of the participants is shown in Table 1.

**Table 1:** Demographic information of the participants in UX2.1^a^.

Gender	Age (years)	Position	Experience (years)	Highest degree or level of school	Competency level in computer
Male	50-59	Physician	24	Doctorate	Advanced
Female	30-39	Associate Director, Clinical Research	14	Doctorate	Intermediate
Male	30-39	Clinical trial Statistician	10	Masters	Advanced

^a^UX: user study.

##### Study Protocol

The study protocol used was as follows:

- Participants were invited via email to be a part of the study. In the email, they were informed that the study would be conducted in-person at the campus for their ease.
- On the day of the study, the participants were briefed about the purpose of the study. The participants were informed that notes would be taken during the discussion.
- They were asked to sign a consent form before starting the study. Once signed, they were asked to complete the demographic information as part of the prestudy questionnaire (I2.1).
- The participants were asked to read through the use cases and provide suggestions on how to improve them. For each use case in the questionnaire (I2.2), the participants were asked to choose their most relevant target user, followed by a descriptive comment justifying their choice. The comments would help in making necessary changes to the use cases based on the recommendations when the use case is relevant. Additionally, they were asked 3 closed-ended questions and 1 open-ended question as described in I2.2.
- The participants were asked to evaluate the system using the heuristic walkthrough method [68]. A heuristic walkthrough is an inspection technique that combines the benefits of heuristic evaluations, cognitive walkthroughs, and usability walkthroughs [68]. It is a 2-step process. First, the participants evaluate the system based on a set of tasks and answer questions for each task based on the use cases 1, 2, and 5 from I2.2. Second, the participants identify the usability problems in the prototype and classify them using Nielsen’s heuristics [69] broken down by types of usability issues. The participants were provided a reporting template form (I2.3) to ease the process. Finally, the participants were asked to complete a poststudy questionnaire (I2.4).

##### Instruments Used

###### Overview

A total of 4 instruments were used in this study, including a prestudy questionnaire (I2.1), a use case questionnaire (I2.2), a usability problem reporting template (I2.3), and a poststudy questionnaire (I2.4). The details of each instrument and the questions included are provided in the following subsections.

###### I2.1: Prestudy Questionnaire

The prestudy questionnaire gathered basic information on demographic and computer skills from the participants. The questions were about gender, age, job position, university/institution/company (if a student/employed), years of experience, nationality, highest degree, and competency level of the computer.

###### I2.2: Use Case Questionnaire

For each use case, the participants were asked to choose the most relevant target user among 3 possible options, that is, “nurse”, “clinician,” and “not relevant”. The participants were further asked to write a descriptive comment justifying their choice. They were also asked 3 closed-ended questions followed by 1 open-ended question. The participants had to choose the best option based on the 5-point Likert scale (1 for “strongly disagree” to 5 for “strongly agree”) for each close-ended question. The open-ended question was to provide comments for the use case. The closed-ended use case questions (UCQs) are reported in Table 2.

**Table 2:** Closed-ended questionnaires I2.2 and I2.4.

Category and code		Text
**I2.2 (Use cases)**		
	UCQ1^a^	It was *simple* to use this system
	UCQ2	I could effectively *complete* the tasks using this system
	UCQ3	I was able to complete the tasks *quickly* using this system
**I2.4 (Overall system)**		
	OSQ1^b^	Overall, it was *easy* to use this system
	OSQ2	It was *simple* to use this system
**I2.4 (Usability)**		
	USBQ1^c^	It was *easy* to learn to use this system
	USBQ2	The information provided with this system was *clear* and easy to understand starting from a search query, navigating by tree keyword levels, up to getting a website description with a link to the targeted website
	USBQ3	It was easy to *find* the information I needed
	USBQ4	The information was effective in helping me *complete* the tasks
	USBQ5	The *organization* of information on the system screens was clear
	USBQ6	I *liked* using the interface of this system
**I2.4 (Usefulness)**		
	USFQ1^d^	This system has all the functions and capabilities I expect it to have, and
	USFQ2	Overall, I am satisfied with this system performance

^a^UCQ: use case question.

^b^OSQ: overall system question.

^c^USB: usability question.

^d^USF: usefulness question.

###### I2.3: Usability Problems Reporting Template

The template provided the participants with an opportunity to report usability problems that need to be fixed in the prototype. For each usability problem, they were asked to provide a solution/recommendation from their perspective. They were also asked to add a severity rating of the problem as 0 for no problem, 1 for cosmetic, 2 for minor, 3 for major, and lastly 4 for catastrophe.

###### I2.4: Poststudy Questionnaire

The questionnaire contained 2 closed-ended and 1 open-ended question about the overall system usage, 6 closed-ended questions for usability, and 2 closed-ended questions on the usefulness of ActiVis. For the closed-ended questions, participants had to choose 1 option based on the 5-point Likert scale (1 for “strongly disagree” to 5 for “strongly agree”). The closed-ended questions in the 3 said categories along with the codes assigned to each question are shown in Table 2.

#### UX2.2: Expert Evaluation 2

##### Overview

A family doctor was invited to evaluate the second prototype (RP2) to realize the tasks of use cases corresponding to that role from the list refined in UX2.1.1. We followed a subset of the protocol used in UX2.1.2.

##### Participant

The study involved a Spanish family doctor visiting Qatar Computing Research Institute (QCRI) during October 2018, as part of his collaboration with a former investigator on this project to give feedback on QCRI’s ongoing research projects in the area of medical/health informatics. This physician was from southern Spain where a large proportion of the population are migrants from the Middle East and North Africa (ie, having Arabic origins).

##### Study Protocol

- The family physician was contacted through email. The participant was invited to take part in the study to share his experience and knowledge, and give feedback on the ActiVis user interface based on 3 use cases refined after UX2.1 that corresponded to the family doctor role (use cases 1, 2, and 5 were selected in Table 3). The participant acknowledged and agreed to be part of the study.
- During the study, the participant was briefed about the purpose of conducting this research and its objectives, and then introduced to the ActiVis user interface. The participant was allowed to have an informal discussion with the researcher to resolve any issues or seek any clarification before they begin the study. Written consent was also taken to be part of the evaluation.
- The participant was informed that notes would be taken throughout the study, the discussion would be audio-recorded, the interaction during the user interface walkthrough of ActiVis would be recorded through a screen recorder application for the analysis as a backup if any point is missed while taking notes.
- The participant was informed to use a think-aloud protocol while exploring ActiVis based on the use cases. This allowed them to say out loud whatever they were thinking about how to perform a task described in each use case on ActiVis.

**Table 3:** Use cases (UX2.1).

Initial description resulting from the analysis of UX1^a^ by the designers and evaluated in UX2.1.1	Target user resulting from UX2.1.1
Use case 1 (Check activity level of a patient): Nurse is at her office; she gets an alert regarding patient sleep quality. Nurse accesses data of the patient; she visualizes the sleep pattern over consecutive days to check how regular it is. She detects irregular sleep time and duration with additional naps on certain days. In particular, she discovers the sleep duration is often short, and the quality of sleep is often poor. She also discovers patient activity is low to moderate.	Nurse/family doctor/clinician researcher
Use case 2 (Comparing activity between weekdays and weekends): Nurse wants to compare the average activity of the patient across weekdays and on weekends. She wants to identify irregular sleep patterns that could cause more fatigue. She discovers longer sleep duration during weekends. Also, notes that naps mostly occur around 4 PM during weekdays and around 12 PM during weekends.	Family doctor/clinician researcher
Use case 3 (Comparing 1 individual before and after intervention): Nurse compares the average activity of the patient at different periods, before and after the intervention, to assess the effectiveness of the intervention. She can see the more regular sleep pattern both during weekdays and weekends after the intervention than before it. She can also compare biometrics such as the normalized BMI and weight, between the 2 periods, and she can identify a loss of weight and decrease of BMI.	Family doctor/clinician researcher
Use case 4 (Comparing 2 individuals [siblings] over a long period): The nurse wants to compare the body metrics and sleep quality of Patient 1 aged 8 years and Patient 2 aged 10 years who are siblings, over a long period to detect a potential family lifestyle issue. The nurse compares the average activity level on weekdays and weekends, and BMI of Patient 1 and Patient 2. She observes that both follow a similar but abnormal pattern of BMI consistent with the average activity level of the corresponding periods, leading to the conclusion that it is a family lifestyle issue.	Clinician researcher
Use case 5 (Comparing an individual to a group): Nurse compares the average level of activity of the patient with the peer group of the same gender. She can see that the patient is among the overweight subgroup, although her average activity level is similar to one of the normal subgroups, leading her to conclude that the patient may have an unbalanced diet or another health issue affecting her weight.	Family doctor/clinician researcher
Use case 6 (Comparing males and females of a group before and after intervention): Nurse compares the average level of activity of 2 subgroups of different genders from a group before and after intervention to assess the effectiveness of the intervention. She can see that males increase their activity level after school during weekdays, while females increased their sleep quality, having a more stable bedtime, especially during weekends. She can also compare biometrics such as the normalized BMI and weight, between the 2 periods and she can identify a loss of weight and decrease of BMI more important for the male group.	Clinician researcher

^a^UX: user study.

##### Instruments Used

This expert evaluation study used 2 of the instruments (I2.1 and I2.2) described in UX2.1.2.

#### UX2.3: Users’ Evaluation Workshop 2 With Nurses

##### Overview

The methods used to conduct this workshop were the same as for UX1. This workshop was conducted with the nursing informatics staff at HMC-Q to evaluate the second prototype (RP2). The workshop was also conducted with the same department and at the same venue as in UX1. It was expected that some of the staff would be the same who attended the first workshop.

The purpose of conducting this workshop was to perform a summative evaluation of the latest version of the prototype and compare it with the Fitbit Dashboard, gather their qualitative feedback, and further improve the user interface.

##### Participants

The recruitment process of the nursing staff was the same as for UX1. A total of 45 participants, including nurses as well as nursing informatics professionals, attended the workshop.

##### Study Protocol

The staff of the nursing informatics department was assigned at random to 1 of 4 tables, where each table could accommodate a maximum of 10 participants. Two groups were randomly chosen and assigned to work with the Fitbit Dashboard, while the remaining 2 groups were assigned to work with the ActiVis Dashboard. All the groups were provided a laptop to explore the assigned dashboard in a web browser using temporary credentials to log-on to the dashboard. Each group was instructed to appoint 1 participant as a *group representative* who would lead the evaluation and inform them about the tasks to be performed. Each group was also instructed to nominate 1 participant as a *group secretary* who would document the entire discussion and problem found as a part of the evaluation. Each group was also given a task-driven walkthrough template.

##### Instruments Used

**Figure 5:**
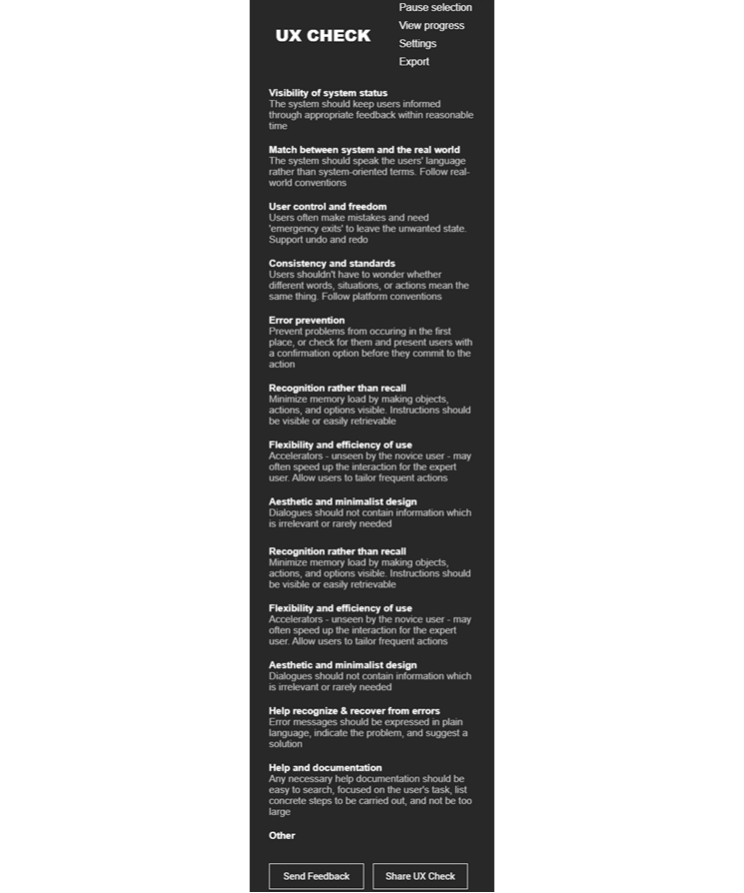
UX Check chrome extension [70] showing Nielsen’s 10 heuristics [69]. UX: User study.

Two instruments were used in this study. These include (1) task-driven walkthrough template, and (2) heuristic evaluation of the dashboard (RP2). The details of each instrument and the questions included are provided in the following subsections. Heuristic evaluation is a usability inspection method that uses evaluators to identify and assess the usability problems in a user interface as a part of the iterative design process. This method relies on the expertise of the domain experts to identify the usability problem in a user interface that needs to be fixed, categorize each identified problem in the heuristics, and rate its severity. The set of 10 heuristics by Nielsen [69] (Figure 5) is the most commonly used in the industry.

###### I4.1: Task-Driven Walkthrough Template

The template contained the following 3 tasks. These 3 tasks were derived from use case 1 (Table 3) proposed after analysis by the designers of the results of the collaborative workshop with nurses (UX1) and validated as a result of UX2.1 with clinicians. Use case 1 is targeted specifically at nurses. These task numbers would be referred to in the results of UX2.3.

- Task 1: Search for the average number of steps for last week.
- Task 2: Search for average active minutes for last month.
- Task 3: Search and describe sleep patterns from May 20 to July 31, 2015.

Each group was asked to brainstorm about the steps needed to complete the task. To guide on how to come up with concrete steps, the following steps were required to complete the first task.

- Enter Patient’s Name/Search in dropdown
- Navigate to Charts
- Observe the particular chart

For each step, the group was asked to answer the following questions:

- Will the user realistically be trying to do this action?
- Is the action visible?
- Will the user recognize the action as being the correct one?
- Will the user understand the feedback/Is the feedback appropriate?

###### I4.2: Heuristic Evaluation of the Dashboard

For the heuristic evaluation, each group was instructed to download and add the “UX Check” [70] extension in the Google Chrome browser. This extension allows an interactive way to identify and describe the usability problems found on the web page. Opening the extension while staying on any page will show the UX Check panel on the left side of the browser as shown in Figure 5.

**Figure 6:**
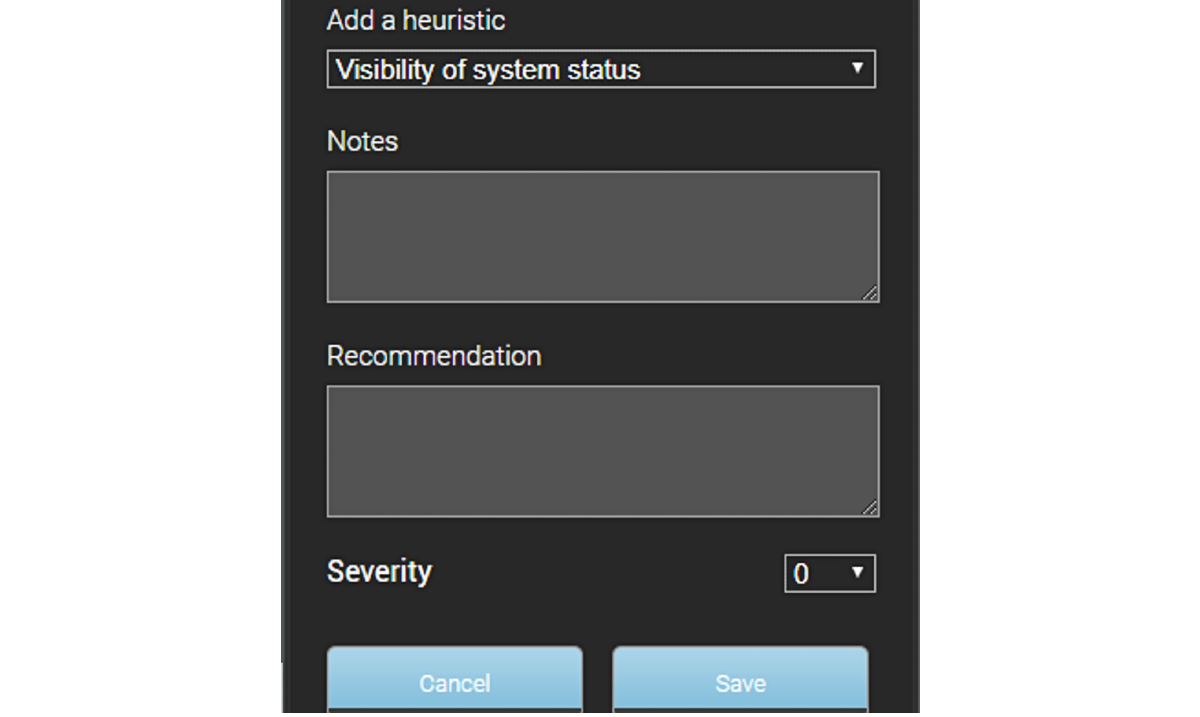
Problems description and recommendation with UX Check [70].

The extension will create the necessary regions that can be selected using a single click of the mouse. Users first need to identify any region that contains the usability problem. Clicking on the region will pop-up the dialog as shown in Figure 6. The pop-up allows users to add the heuristic problem, problem description in the form of notes, possible recommendations to fix the problem from their perspective, and lastly the severity rating. The numbers and associated description of the rating are discussed in the “Results” section. Users can save the problem for reporting or cancel their actions. The extension provides a facility for users to view all the identified problems by clicking on the “View progress” link in the pop-up shown on the left side of the web browser. They can export all the problems identified to a Microsoft Word Document by clicking on the “Export” link.

##### Ethics Approval

The ethical approval was sought from the Qatar Biomedical Research Institute Institutional Review Board of Hamad Bin Khalifa University, Qatar, before conducting this research (QBRI-IRB 2018-019). The health care professionals as potential users were involved in all the studies as part of this research. Following the cycles of user-centered design, each study on a prototype with health care professionals provided feedback, which was used as a requirement to design an improved version as the next prototype.

## Results

### RP1: Visual Analytic Tool for Actigraphy Sensor Data

**Figure 7:**
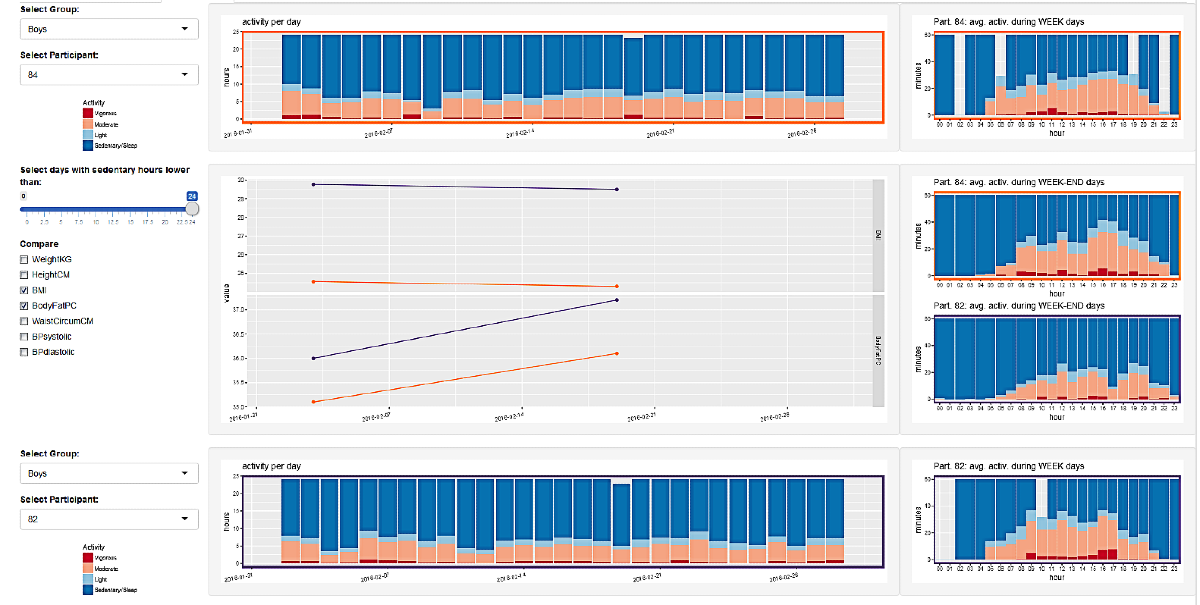
First version (RP1) of the ActiVis tool: the left panel is used for patient and group data selection, and filtering on body metrics and activity features; the right panel shows the resulting display for overview and patient/patient and patient/group comparison.

We developed 2 versions of the ActiVis interface. The first version (RP1) is shown in Figure 7 and was used in UX1. It was the result of the previous analysis not reported in this study. We proposed a visualization focused on 2 generic tasks: patient overview and comparison, inspired from the discussion with a previous “obesity camp” project participants, and based on the available data [62,63].

Data are body metrics (eg, BMI, weight, height) measured at regular intervals during the obesity camp, together with minute-based activity recordings from wearable accelerometers.

**Figure 8:**
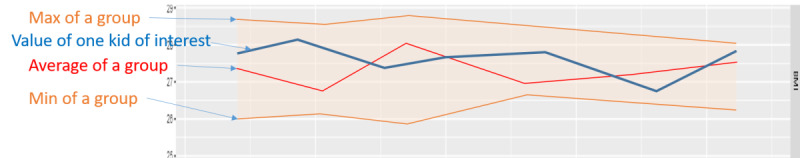
Details of the line chart: this chart shows the evolution of the body metric of interest (vertical axis) through time (horizontal axis) for a single patient (blue line), and a group of patients showing its minimum (orange bottom line), maximum (orange top line), and average (red line) values.

**Figure 9:**
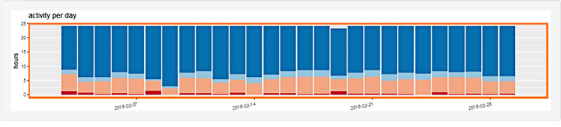
Details of the bar chart: Each vertical bar codes for the breakdown of activity levels per day, for sleep (dark blue), sedentary (light blue), moderate (orange), and vigorous (red) activity levels.

The interface supports an overview and comparison between the data of 2 patients, or 1 patient and a group of patients. The left panel allows selecting the patient and the body metrics features to be displayed. The right panel shows multiple line charts coding for each of the selected features through time coded on the horizontal axis. Color of the line (orange or purple) represents the selected patient or group (Figure 8). The top and bottom rows show bar charts representing the breakdown of activity levels averaged per day for the corresponding patient or group (orange or purple color of the frame; see details in Figure 9). The rightmost views show bar charts averaging the activity level per hour across the selected time window, during weekdays (first and fourth rows) and weekend days (second and third rows). The selection is done by a range selection on the central bar charts and all charts are cross-linked to focus on the same period.

### UX1: Users’ Evaluation Workshop 1 With Nurses

Nurses have various goals, challenges, and frustrations; however, the results showed that they are mainly concerned about patients’ awareness of their health condition and ways to monitor patients between visits. They particularly need to keep track of patients’ metrics, activity levels, and dietary habits so that they can contact the patients to guide them or remind them about what they have to do as per their activity prescription. Regarding the use of technology, some nurses raised literacy issues and others highlighted accessibility and security concerns.

Nurses highlighted that mobile health (mHealth) apps are an effective means to influence patients’ lifestyles. The most desirable functionalities are activity tracking, dietary advice, and patient education. Including a chat service to facilitate patient-nurse communication is also a viable functionality. Social networking with family and friends is crucial to encourage patients to improve their lifestyles. Interactivity features such as gamification and rewarding achievements were identified as potential ways to motivate patients. Enabling interaction with the app and eliciting patients’ feedback facilitate tailoring contents to suit patient needs.

Outcomes of the workshop showed that recent developments in mHealth apps meet the needs and expectations of their potential users. This is consistent with the latest research findings that confirmed the popularity of mHealth apps (eg, [36]).

The analysis of the workshop usage scenarios led us to design 6 use cases reported in the left-side column of Table 3.

### RP2: Research Prototype 2

**Figure 10:**
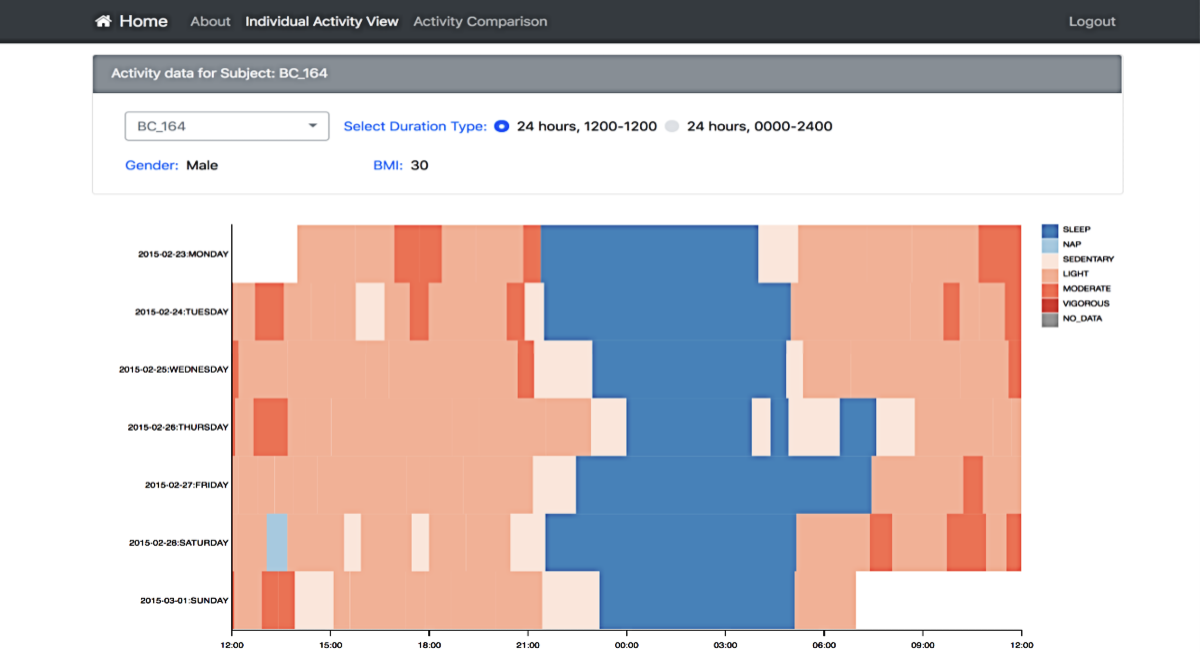
This view supports Use Case 1: Check activity level of a patient. It shows the weekly activity of a patient broken down by day. Each row is a day, and the x-axis shows the hours from noon to noon to focus on weekly patterns of sleep (blue). The user can switch the view (top radio button) to span from midnight-to-midnight range and focus on daily activity level (reddish color). This view gives more details of each day and night, allows a side-by-side comparison, and supports the user in detecting activity patterns across several days.

**Figure 11:**
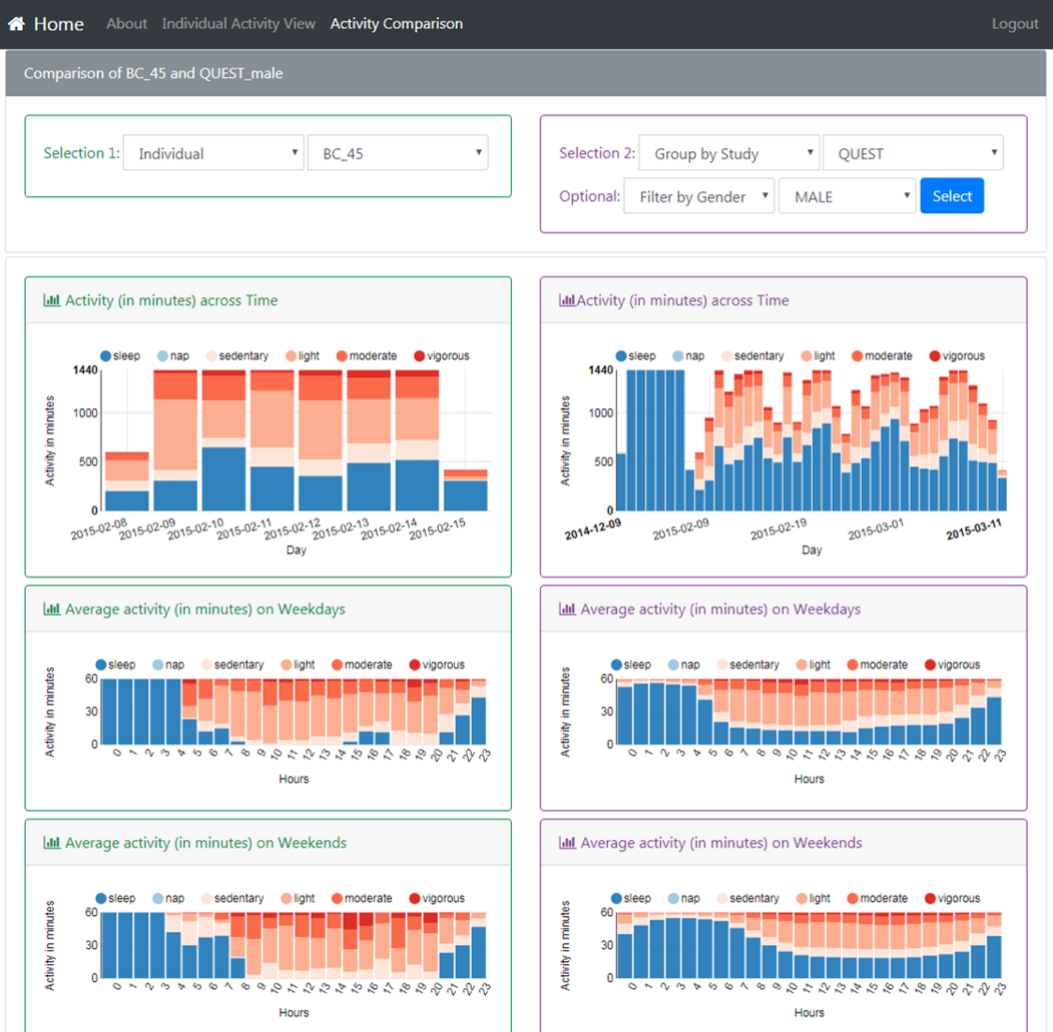
This view supports the qualitative pattern analysis described in Use Cases 2, 3, 4, 5, and 6. It shows a filter (top) to enable the comparison of average weekly activity between a patient or a group (left column) to another patient or a reference group (right).

**Figure 12:**
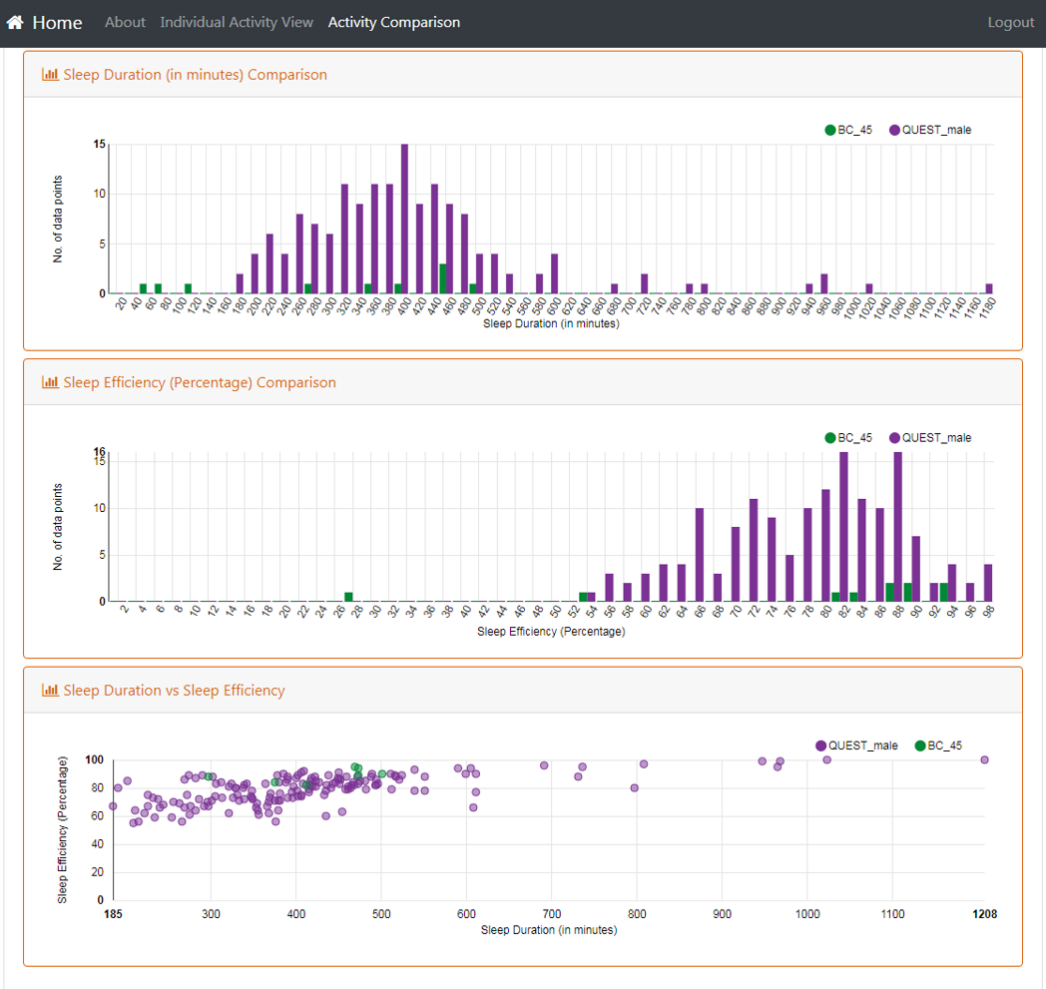
This view supports the quantitative pattern analysis described in Use Cases 2, 3, 4, 5, and 6. It shows the quantitative distribution of two groups of patients along different dimensions as histograms (top two rows) or combined as a color-coded scatterplot (bottom row).

Figures 10-12 show the resulting interface to support the use cases detailed in Table 3. The interface now has 3 different views to support detailed activity analysis of a patient (use case 1) in Figure 10, qualitative comparison of average activities between patients and groups of patients (use cases 2-6) in Figure 11, and quantitative analysis of the same cases in Figure 12.

### UX2.1: Expert Evaluation 1

#### UX2.1.1: Study 1: Use Case Questionnaire

None of the participants selected an option of “Not relevant,” so all the use cases were retained and modified based on the participants’ recommendations.

Table 3 presents 6 use cases resulting from our analysis of UX1, and their reassignment to the correct target user based on the feedback of the participants in UX2.1.1.

The discussion of the results with the participants led us to further distinguish between nurses (use case 1), family doctors (use cases 1, 2, 3, and 5), and clinician researchers (all use cases) types of users. Indeed, the role of a nurse is to observe that a prescribed activity level is correctly followed by the patients to give them reminders if needed, and to notice possible anomalies to report to the doctor, both tasks falling under use case 1. The role of a family doctor is to recommend treatment to the patient. In addition to realizing the tasks assigned to a nurse, the doctor can compare activities or biometrics of a patient between 2 periods (use case 2) to spot differences and recommend a corrective intervention to the patient. The doctors can also control the effect of their prescribed intervention by comparing activity levels and other biometrics before and after it took place (use case 3). At last, the family doctor can compare the patient with statistics derived from groups of patients with similar attributes (age, gender, BMI, or health condition; use case 5). Both nurses and doctors are focused on a single patient at a time. Finally, the clinician researcher focuses on observing trends and patterns within and between cohorts of patients (use case 6), generating knowledge that can guide the family doctors to address the health issue of a specific patient. The clinician researcher can also study more specific cases comparing them over a long period (use case 4) and in general conduct all the other tasks assigned to doctors and nurses for specific patients.

Following this refined assignment, we selected use cases 1, 2, and 5 for further summative evaluation in UX2.1.2 and UX2.2 with the family doctor, because use cases 2 and 3 involved similar tasks. We also focused strictly on use case 1 for the summative evaluation with nurses in UX2.3 as it was the only use case targeted to them.

Table 4 presents the cumulative responses related to the use case questionnaire (I2.2). The first column presents the 3 use cases used in the expert evaluation study (see Table 3 for the full forms of mentioned use cases), the short-form of 3 questions asked for each use case is presented in the subcolumn (see I2.2 and Table 2 for the full form of each question), while the remaining columns contain the cumulative responses in terms of “strongly disagree”, “disagree”, “neutral”, “agree”, and “strongly agree.” Because 3 participants took part in the study, the maximum number of responses is less than or equal to 3. For each use case, the participants found that they were able to effectively, quickly, and efficiently complete the tasks using RP2.

**Table 4:** Cumulative responses of the participants for use cases 1, 2, and 5.

Use case and usability criterion		Strongly disagree	Disagree	Neutral	Agree	Strongly agree
**1**						
	UCQ1^a^	0	0	0	2	1
	UCQ2	0	0	0	2	1
	UCQ3	0	0	0	2	1
**2**						
	UCQ1	0	0	0	2	1
	UCQ2	0	0	0	1	2
	UCQ3	0	0	0	1	2
**5**						
	UCQ1	0	0	1	1	1
	UCQ2	0	0	1	1	1
	UCQ3	0	0	0	2	1

^a^UCQ: user case question.

#### UX2.1.2: Study 2

##### Usability Problems Reporting

The descriptive comments provided by the participants as part of open-ended questions are presented in Multimedia Appendix 1. It is to be noted that minor changes were incorporated in the user interface of RP2 based on the participants’ comments; therefore, no new RP was produced.

##### Poststudy Questionnaire

Table 5 presents the cumulative responses of the overall system, usability, and usefulness from the participant’s point of view as a part of the poststudy questionnaire (see I2.4 for the questions based on the codes used in the subcolumn) using a “clustered column chart.” The format of Table 5 is similar to that of Table 4.

Table 5 shows the usefulness of the system from the participants’ point of view. The participants found that the system had all the functions and capabilities they expected it to have, and they were satisfied with the performance of this system.

In terms of the overall system, Table 5 shows that the participants found that the system was easy and simple to use.

In terms of the usability of the system, Table 5 shows that the participants found that the system was easy to learn, the information provided was clear and easy to understand, the information needed was easy to find, information was effective to complete the tasks, organization of information across the screens was clear, and lastly, they liked using the interface of this system.

**Table 5:** Cumulative responses of the participants UX2.1.2.

Category and code			Strongly disagree		Disagree		Neutral		Agree		Strongly agree
**Overall system**											
	OSQ1^a^	0		0		0		1		2	
	OSQ2	0		0		0		2		1	
**Usability**											
	USBQ1^b^	0		0		0		1		2	
	USBQ2	0		0		0		2		1	
	USBQ3	0		0		1		0		2	
	USBQ4	0		0		0		1		2	
	USBQ5	0		0		0		1		2	
	USBQ6	0		0		0		1		2	
**Usefulness**											
	USFQ1^c^	0		0		0		2		1	
	USFQ2	0		0		1		1		1	

^a^OSQ: overall system question.

^b^USB: usability question.

^c^USF: usefulness question.

### UX2.2: Expert Evaluation 2

The audio-taped RP2 interface walkthrough was analyzed. The problems identified and the recommendations provided by the participant evaluation based on the given use cases are presented in Multimedia Appendix 2. Both problems and recommendations were communicated to the engineers to incorporate necessary changes in ActiVis RP2, leading to minor changes in the user interface of ActiVis RP2 used for the UX2.3.

### UX2.3: Users’ Evaluation Workshop 2 With Nurses

#### Fitbit Dashboard

##### Overview

Table 6 shows the cumulative number of “yes” and “no” against each question for all the steps required to complete tasks 1, 2, and 3 (see I4.1 for the task details) by all the groups using the Fitbit Dashboard. If the answer to any question is “yes,” then it means the group mutually agreed to the statement; however, if an answer to any question is “no,” then it shows the disagreement. In the latter case, they were instructed to add more description so that the problem can be rectified in the user interface. However, during the analysis of the filled templates returned by the groups, it was found that some of the groups also commented when their answer was “yes.” Such comments mainly reflected the minor changes recommended by the group despite an agreement to the question.

**Table 6:** Cumulative number of responses against each question for all the steps required to complete tasks using the Fitbit Dashboard.

Fitbit	Questions							
	Q1: Will the user realistically be trying to do this action?		Q2: Is the action visible?		Q3: Will user recognize the action as being the correct one?		Q4: Will the user understand the feedback/is the feedback appropriate?	
	Yes	No	Yes	No	Yes	No	Yes	No
Task 1: 6 steps	6	0	5	1	5	1	4	2
Task 2: 4 steps	4	0	4	0	4	0	4	0
Task 3: 4 steps	4	0	4	0	4	0	4	0

The results for each task are as follows:

##### Task 1

For all the steps in Q1, the participants were willing to perform an action. For most of the steps (5/6) in Q2 and Q3, the participants found that the action was visible, and they could recognize that the action performed was the correct one. For 4/6 steps in Q4, the participants found that they were able to understand the feedback, or that the feedback was appropriate.

##### Tasks 2 and 3

For all the steps (4/4), the participants were willing to perform an action, found that the action was visible, that they recognized that the action performed was the correct one, and that the feedback given toward the end of the task was understandable or appropriate.

#### ActiVis Dashboard

##### Overview

Table 7 shows the cumulative number of “yes” and “no” against each question for all the steps required to complete tasks 1, 2, and 3 by all the groups using the ActiVis Dashboard.

The format of Table 7 is similar to that of Table 6. The results for each task are as described in the following sections.

**Table 7:** Cumulative number of responses against each question for all the steps required to complete tasks using the ActiVis Dashboard.

ActiVis	Questions							
	Q1: Will the user realistically be trying to do this action?		Q2: Is the action visible?		Q3: Will user recognize the action as being the correct one?		Q4: Will the user understand the feedback/Is the feedback appropriate?	
	Yes	No	Yes	No	Yes	No	Yes	No
Task 1: 6 steps	5	1	6	0	4	2	3	3
Task 2: 6 steps	5	1	3	3	4	2	4	2
Task 3: 6 steps	4	2	5	1	5	1	6	0

##### Task 1

For most of the steps (5/6) in Q1, the participants were willing to perform an action, for all the steps (6/6) in Q2, the participants found that the action was visible. For 4/6 steps in Q3, the participants were able to recognize that the action performed was the correct one. However, for 3/6 steps in Q4, the participants had mixed opinions; for half of the steps, they found that they were either unable to understand the feedback, or that the feedback was inappropriate, while for the remaining steps, they found that they were able to understand the feedback, or that the feedback was appropriate.

##### Task 2

For most of the steps (3/4) in Q1, the participants were willing to perform an action; however, for 3/5 steps in Q2, the participants found that the action was not visible. For 2/4 steps in Q3, the participants had mixed opinions. For half of the steps, some participants found that they were able to recognize the action performed, while the other participants found that they were unable to recognize the action performed. Similarly, a mixed opinion was also found for Q4 (2/4 steps). For half of the steps, some participants found that they were able to understand the feedback given toward the end of the task, while the other participants found that they were unable to understand the feedback given at the end of the task.

##### Task 3

For 3/5 steps in Q1, the participants were willing to perform an action, for 4/5 steps in Q2 and Q3 each, the participants found that the action was visible and that they recognized that action performed was the correct one. For all the steps, the participants found that the feedback given after the task was performed was understandable or appropriate.

#### Heuristic Evaluation of the Interfaces

**Figure 13:**
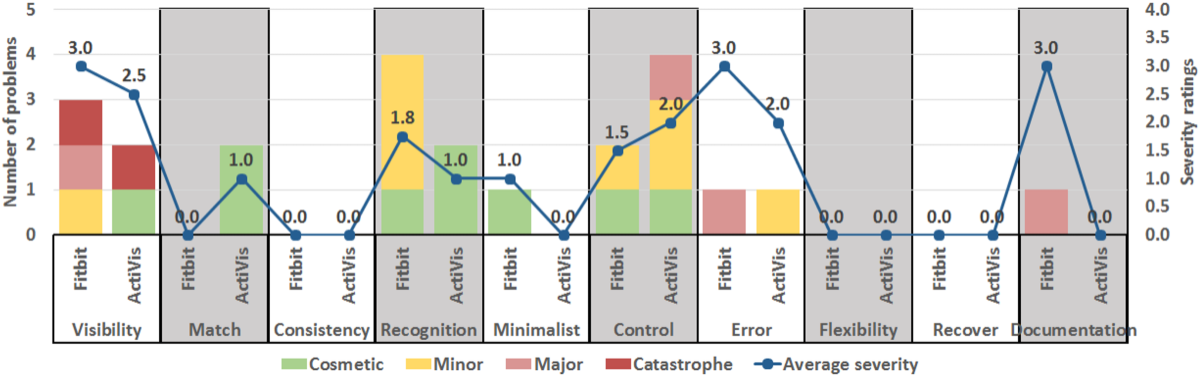
Usability problems identified in Fitbit and ActiVis dashboards.

Figure 13 shows the number of usability problems found and the average severity ratings of the identified problems in the Fitbit Dashboard and the ActiVis Dashboard, respectively, using Nielsen’s 10 heuristics. The “stacked columns” represent the “number of usability problems” (left vertical scale), whereas the “line with markers” represents the “average severity rating of the identified problems” (right vertical scale). Each stack column shows the number of usability problems found based on the 4 severity ratings, that is, cosmetic, minor, major, and catastrophic. The axis on the left-hand side is known as the primary axis and it is related to the “stacked columns,” whereas the axis on the right-hand side is known as the secondary axis and is related to the “line with markers.”

A total of 11 usability problems were identified in each of the 2 dashboards (ie, Fitbit and ActiVis). The analysis of the results in terms of the number of usability problems found in Fitbit shows that the recognition heuristic (n=4) was the more commonly broken heuristic, followed by the visibility and control heuristics (n=2 each). Similarly, the analysis of the results in terms of the number of usability problems found in ActiVis shows that the control heuristic (n=4) was the more commonly broken heuristic, followed by the visibility, match, and recognition heuristics (n=2 each).

The analysis of the results in terms of the average severity rating shows that the majority of problems identified are minor.

The number of usability problems identified and their severity rating provided by the participants for the Fitbit Dashboard and the ActiVis Dashboard were the same. However, the Fitbit Dashboard has more severe issues than the ActiVis Dashboard in terms of visibility, recognition, error, and documentation. Still, ActiVis needs improvement compared with Fitbit in terms of control and match, and to solve the catastrophic visibility issue identified.

### RP3: Research Prototype 3

The work on this project is still ongoing. The 3 UX (UX2.1, UX2.2, and UX2.3) of the second ActiVis prototype (RP2) led to new and updated requirements for RP3. Since the last study, the work on this interface has been organized in 2 different branches. The research effort specific to the visualization interface has been split between the different types of users (nurses described in use case 1; family doctors in use cases 2, 3, and 5; and clinician researchers in use cases 4 and 6) with specific charts and interactions but with a common core of data processing functions. The developed visualization prototypes are planned to be integrated into a platform able to read data from different wearable devices available on the market, and integrated into a clinic environment. User evaluations will continue as part of the user-centered design and PD cycles.

## Discussion

### Principal Findings

The key finding from these PD studies is the derivation from post hoc analysis of nurses’ workshop, and the validation by 2 physicians, 1 clinician researcher, and 1 clinician statisticians of the 6 use cases to analyze wearable data for health care professionals. These use cases are assigned to specific user roles: nurses, family doctors, and clinician researchers. They will facilitate the design and development of new data analytics and visualization interfaces to support the particular needs of these users.

### UX1

During the PD workshop with nurses evoking their work and relations with patients and other health care professionals, we could not identify specific cultural needs in terms of the visualization of wearable data for health care professionals. Some of the persona and usage scenarios were obviously representative of the local Arabic culture by design, and it is also well-known that particular customs such as prayer times and fasting during the Ramadan Holy month can impact people’s patterns of physical activities, sleep, and diet, but none of these aspects finally influenced the more technical use cases we derived from these discussions. The use cases we propose ended up being culturally agnostic (Table 3).

### UX2.3

The final evaluation comparing Fitbit and ActiVis dashboards showed there is ample room for improvement even in existing interfaces such as Fitbit, widely available for the general public. We only evaluated use case 1 specific to nurses and already identified some major and catastrophic problems, with severe ratings being more frequent with Fitbit than with ActiVis. Although Fitbit was not necessarily designed to support this use case, it shows that we cannot simply reuse available interfaces to support end users in the best way. Supporting statistical and visual analyses of wearable data from cohorts of patients as stated in use cases targeted at clinician researchers are not optimal or even possible with existing visualization tools and will deserve further investigations.

In general, this project also showed how conducting PD is necessary but still challenging. It has been difficult to plan several of the studies in advance. The use of the opportunistic approach allowed us to use the available local health care professionals throughout the design, development, and validation of RPs presented in this paper. Qatar is a country where 90% of the population are expatriates mixing Western, Asian, and Muslim cultures. Because of the heterogeneous culture and origin of the population, it is challenging to study the levels of health awareness in Qatar [71]. Nevertheless, this is crucial to understand to develop efficient health-targeted visualizations. The population diversity also allowed us to get feedback from non-Qatari, non-Muslim users too. Opening to a wide range of cultures in the same place is of interest to understand what is common or specific to these end users. Although the interface for health care professionals is not impacted by local culture, we know from a previous study [72] that the interfaces involving the patients themselves will need specific care of their local particular health conditions (eg, diabetes or obesity) and Muslim culture (Ramadan Holy month effect on diet, sleep, and physical activity).

### Limitations

The study has several limitations. First, a specific set of methods from the user-centered design and PD methodologies was used. Second, the studies were conducted with a selected list of institutions and their experts as participants. Third, several participants were used in each study that was mainly dependent on multiple factors, including availability based on their routine clinical appointments, meetings, and teaching. Fourth, Nielsen’s heuristics were used to diagnose user problems in the prototype that need to be fixed. All these constraints could affect the generalizability of the results. For future studies, we seek higher diversity and a higher number of participants, and domain-specific heuristics to get more generalizable findings.

### Conclusion

This paper shows how the use of PD and user-centered design allowed the development of a visualization interface supporting the real needs of health care professionals in Qatar. Although Qatar is an oil-based economy that nurtures a rich multicultural environment, the use cases we derived from the PD studies happen to be culturally agnostic. We hope these use cases will serve to design future visualization and analytic systems optimized to support the needs specific to nurses, family doctors, and clinician researchers, beyond existing dashboards designed primarily for the general public. This work is still ongoing. A cluster project has now started that is funded under the Qatar National Research Fund [73] and will support further development and integration of these visualizations in a clinical setting to help clinician researchers, doctors, and nurses improve the health of Qatari citizens and residents.

## Appendix

Multimedia Appendix 1 Expert comments.

Multimedia Appendix 2 Expert recommendations.

## Abbreviations

HMC-Q: Hamad Medical Corporation-Qatar
PD: participatory design
QCRI: Qatar Computing Research Institute
RP: research prototype
UX: user study
WCM-Q: Weill Cornell Medicine - Qatar
WHO: World Health Organization
