# Supplementary material for: A Participatory Design Approach to Develop Visualization of Wearable Actigraphy Data for Health Care Professionals: Case Study in Qatar
Source: JMIR Hum Factors. 2022 Apr 8;9(2):e25880. doi: 10.2196/25880 (PMC9034423; doi:10.2196/25880)
Supplement: Multimedia Appendix 1 [file humanfactors_v9i2e25880_app1.docx]

Table A-1: Expert comments

| **Open-ended question for** | **Descriptive comment** |
| --- | --- |
| Use case 1: | Compare with average and 2sd of the larger population |
|  | The activity was easy to visualize. Need to define what constitutes a nap. |
|  | Very good visuals and easy to understand |
| Use case 2: | Duration of naps needs to be defined |
|  | Visualization of weekday vs weekend data is nice. |
|  | There was no indication as to what a weekday or weekend was |
| Use case 5: | Average and SD of the group on individual's graph; Individual to be pointed out on group figure; axes to match; ability to select exactly what I need to look at rather than eliminate |
|  | This feature is really powerful. It could, however, benefit from ensuring scales are the same for easier comparison and also matching the group data e.g. by day rather than actual date so the number of bars is ideally ordered the same (day of the week instead of day 1, day 2 etc.  It would also be good to be able to filter further - especially by age as this is an important factor. |
|  | Comparing 7 days with 30 days is difficult |
| Overall comments: | The system is user-friendly. The only task that was not immediately obvious was re-selecting the individual as the first step in the comparison screen. |
