# Supplementary material for: A Participatory Design Approach to Develop Visualization of Wearable Actigraphy Data for Health Care Professionals: Case Study in Qatar
Source: JMIR Hum Factors. 2022 Apr 8;9(2):e25880. doi: 10.2196/25880 (PMC9034423; doi:10.2196/25880)
Supplement: Multimedia Appendix 2 [file humanfactors_v9i2e25880_app2.docx]

Table A-2: Expert recommendations

| **Use case** | **Problem description** | **Recommendation** |
| --- | --- | --- |
| 1 | Sleep activity creates confusion with other activities | There should be an issue for a user to select what to see and what to hide so that they can easily concentrate on the data |
| 1 | It's difficult to visit individual sleep bars in the graph to find out whether sleep quality is poor or good | Such information should be communicated straightaway to the user |
| 1 | It was difficult to find out how sleep duration is often short as asked in use case 1. | There is only one sleep bar so the assumption is data is stored somewhere but not visible enough for identification. |
| 1 | There was no option to separate graphs of physical activities with sleep. | There should be a way for the user to separate the graphs shown. |
| 1 | When all days are represented in the same color, it's difficult to differentiate between the weekdays and the weekend. | It would be good to show them in different colors. The colors for the weekend should be different so that one can easily identify that they are weekends. |
| 1 | The expert used the “Activity comparison” to work on this case as it was not easy for him to work in the “Individual activity view”. | The look and interaction of both screens that show “Activity comparison” and “Individual activity view” should be the same. The information would be easier to see and interpret if the graphs are shown the way they are shown in the "activity comparison". |
| 2 | Still, the descriptive information shown as a tooltip was difficult to interpret. Also, in the "activity comparison" view, the information is shown in terms of the dates, but it is not known which date is weekday and weekend. | Show all the dates and weekends should be shown in a different color based on the locale. |
| 2 | The interpretation in terms of the minutes is difficult. | It would have been better if there was an option to view/change in terms of "hours, minutes, etc." |
| 2 | The comparison between the two graphs of activity during weekdays and weekends was difficult in terms of visual interpretation. |  |
| 2 | It’s difficult to interpret different types of exercise. | They should be grouped together. If more interested, then one can go further to see all types of exercises (light, moderate, etc.) |
| 2 | The Time is shown in hours rather than the minutes | There should be an option for the user to change the view from hours to minutes and vice versa. |
| 5 | It was not clear which group to be compared? The options in the list are not easy to read. For instance, group by the study shows “MASSES, QUEST, hchc”. The expert was not sure what these are | The names of the groups should be user-friendly and easy to read. |
| 5 | When we don’t know the gender of an individual (male or female), selecting an option to compare with the male and female is very difficult |  |
| 5 | The data for an individual was shown for more number days than the group which also makes comparison difficult. | There should be consistency in terms of days and weeks shown in both graphs. It would have been much easier to see through different lines as shown in FitBit |
